# Haze Influencing Factors: A Data Envelopment Analysis Approach

**DOI:** 10.3390/ijerph16060914

**Published:** 2019-03-14

**Authors:** Yi Zhou, Lianshui Li, Ruiling Sun, Zaiwu Gong, Mingguo Bai, Guo Wei

**Affiliations:** 1 School of Applied Meteorology, Nanjing University of Information Science and Technology, Nanjing 210044, China; 20171103093@nuist.edu.cn (Y.Z.); 20171103091@nuist.edu.cn (R.S.); 2 School of Management Science and Engineering, Nanjing University of Information Science and Technology, Nanjing 210044, China; llsh@nuist.edu.cn; 3 School of Business, Anhui University of Technology, Maanshan 243032, China; baimingguo@163.com; 4 Department of Mathematics and Computer Science, University of North Carolina at Pembroke, Pembroke, NC 28372, USA; guo.wei@uncp.edu

**Keywords:** PM_2.5_, human activities, meteorological factors, chance constrained stochastic DEA

## Abstract

This paper investigates the meteorological factors and human activities that influence PM_2.5_ pollution by employing the data envelopment analysis (DEA) approach to a chance constrained stochastic optimization problem. This approach has the two advantages of admitting random input and output, and allowing the evaluation unit to exceed the front edge under the given probability constraint. Furthermore, by utilizing the meteorological observation data incorporated with the economic and social data for Jiangsu Province, the chance constrained stochastic DEA model was solved to explore the relationship between the meteorological elements and human activities and PM_2.5_ pollution. The results are summarized by the following: (1) Among all five primary indexes, social progress, energy use and transportation are the most significant for PM_2.5_ pollution. (2) Among our selected 14 secondary indexes, coal consumption, population density and civil car ownership account for a major portion of PM_2.5_ pollution. (3) Human activities are the main factor producing PM_2.5_ pollution. While some meteorological elements generate PM_2.5_ pollution, some act as influencing factors on the migration of PM_2.5_ pollution. These findings can provide a reference for the government to formulate appropriate policies to reduce PM_2.5_ emissions and for the communities to develop effective strategies to eliminate PM_2.5_ pollution.

## 1. Introduction

Recognizing the key influencing factors of haze is critically important for haze control. The purpose of this study is to explore contributing factors related to human activities or meteorological events.

Haze not only pollutes the ecological environment, but also seriously affects the physical and mental health of humans. Low visibility in haze weather could easily lead to traffic accidents, endangering human life and property [1]. The particulate pollutants in haze can cause great harm to humans and the toxic effect is mainly ascribed to PM_2.5_, defined as fine particulate matter with a diameter smaller than 2.5 μm [2]. Due to their size, PM_2.5_ particles can penetrate into the respiratory tract and reach the alveoli [3], and can even enter the systemic circulation and affect other organs [4]. PM_2.5_ may also directly affect the cardiovascular system by entering into the systemic circulation and causing cardiovascular dysfunction [5]. They can also cause depression and other mental illnesses [6]. As PM_2.5_ is the main component of haze and causes great harm to humans, the key to control haze pollution is to decrease PM_2.5_ pollution. Therefore, this paper focuses on investigating the influencing factors of PM_2.5_ pollution.

In recent years, the haze condition in China has become so significant that the problem has triggered a warning to the government, the communities, and all citizens. The annual average concentration of PM_2.5_ from 2013 to 2016 is as high as 57.75 μg/m^3^ in China [7], which is over six times higher than that of the United States (8.47 μg/m^3^ [8]) or 5 times higher than that of the WHO standard (10 μg/m^3^).

### 1.1. Human Activities

Many scholars have conducted various studies on the causes and mechanisms of haze, hoping to provide scientific advice for the government to prevent and control haze [9,10,11]. It is known that human activities, such as industrial pollution and fuel combustion, automobile exhaust, construction dust, road dust and industrial dust, contribute substantially to haze pollution. Taking Beijing as an example, in 2017, vehicle exhaust, coal combustion, industrial production and dust emission were the main sources of PM_2.5_, accounting for 31.1%, 22.4%, 18.1% and 14.3%, respectively [12].

Direct emissions mainly come from various types of combustion, including fossil fuel (coal, oil, natural gas) combustion and biomass burning (straw, firewood). Coal accounts for a large proportion of China’s energy consumption structure. In 2017, coal consumption accounted for 60.4% of total energy consumption [13].

Haze pollution caused by coal combustion in China can be divided into two types by scale: one is large-scale high-density coal combustion such as power plants and iron and steel enterprises. You and Xu [14] along with Tang et al. [15] found that coal combustion is the main source of particulate pollution in China.

The other is small-scale coal combustion, such as household coal combustion, which is widely distributed and easily overlooked. Cheng et al. [16] pointed out that the actual coal consumption by residents is 4.6 times that of the amount reported in the statistical yearbook for that year, and hence household coal combustion should not be ignored. The studies by Xue et al. [17] and Zhang et al. [18] indicated that PM_2.5_ emissions from the residents of the Beijing-Tianjin-Hebei region accounted for nearly 50% of the total PM_2.5_ emissions and is exhibiting an upward trend.

Pollutants that can be turned to PM_2.5_ pollution include nitrogen oxides, sulfur dioxide and volatile organic compounds. Vehicle exhaust is a main source of this kind of PM_2.5_ pollution. Ming et al. [19], Gao et al. [20] and Yang et al. [21] pointed out that the increase in the number of cars has caused a significant increase in haze pollution. Gao et al. [22] assessed the health damage caused by vehicle exhaust. Goel and Kumar [23] studied the exposure to vehicle exhaust under different traffic conditions, and their findings show that PM_2.5_ pollution caused by vehicle exhaust when traffic jams is more serious than when traffic flow is smooth.

Construction dust, road dust and industrial dust are also important sources of PM_2.5_. Kinsey et al. [24] and Hassan et al. [25] found that the dissipation of dust during construction is one major source of PM emissions. Zhao et al. [26] pointed out that high-intensity economic activities and large-scale infrastructure construction are reasons for the increase in PM_2.5_ concentration in China. Lin and Zhu [27] and Wu et al. [28] found that the concentration of PM_2.5_ is closely related to the urbanization process in China. Zhou et al. [29] showed that there was a significant positive relation between the emission of industrial dust and the concentration of PM_2.5_.

### 1.2. Meteorological Factors

Some scholars suggest that meteorological events are natural causes of haze formation. Studies found that meteorological factors related to haze include relative humidity, wind speed, and precipitation. The studies by Fu et al. [30], Chambers et al. [31], Yang et al. [32] and Li et al. [33] indicated that relative humidity and wind speed have a great relationship to haze pollution. Guo et al. [34] found that the washing effect of precipitation can significantly reduce haze pollution.

While meteorological factors are natural causes or objective factors for the formation of haze, human activities account for a significant part of the pollution, especially for PM_2.5_ pollution. PM_2.5_ pollution conditions are the combined effects of meteorological and human factors. However, in the past, only a few studies have considered the combined effects of meteorological conditions and human activities on haze formation.

### 1.3. Our Approach

Jiangsu is an important industrial province in China, with a GDP (Gross Domestic Product) of 8.59 trillion RMB in 2017, which is about 10.03% of China’s total GDP or the national GDP of Russia (8.51 trillion RMB) [35]. With the fast development of industrialization and urbanization, people there are also facing heavy pollutant emissions, and haze and fog have become a serious concern for the residents and a burden on the public. Under China’s new standard economic system, people urgently demand air quality improvement. As the key task of ecological environment governance, PM_2.5_ pollution prevention and treatment have become a critical task to win “the battle for blue skies”. Differentiating the impact of different factors on PM_2.5_ pollution in Jiangsu Province can provide more precise information for the government and communities to design suitable policies and care strategies.

Meteorological factors and human reasons will be taken as the influencing factors of PM_2.5_ pollution (Section 3). Through related literature analyses, human factors are divided into four major categories: (1) Social progress: including the impact of urbanization, housing construction and demolition, and population growth. (2) Transportation: vehicle exhaust emissions. (3) Energy use: fossil fuel combustion. (4) Environmental protection: human efforts to reduce pollution. Because the social and economic data are described by years, but the meteorological data are provided by units of hours or days, we face the problem of the time scale not being uniform when the relevant data is processed.

DEA is a linear programming methodology to evaluate the efficiency of multiple decision-making units (DMUs) when the production process presents a structure of multiple inputs and outputs [36]. This method is not required to determine the explicit expression of the relationship between input and output variables, which eliminates many subjective factors, and hence, has strong objectivity. Therefore, the DEA method is widely used in efficiency evaluation [37]. However, traditional DEA does not consider the random errors of input and output variables, such as measurement error and data noise [38,39], which are unavoidable in data collection [40].

In this paper, the stochastic data envelopment analysis model is applied to resolve this problem. When compared with traditional DEA, chance constrained stochastic DEA has two advantages: admitting random input and output, and allowing the evaluation unit to exceed the front edge under the given probability constraint [41,42]. We study the coupling effect of multiple factors in the effort to identify the influencing factors of PM_2.5_ pollution in Jiangsu province.

The remainder of the article is organized as follows: Section 2 presents the traditional DEA method and chance constrained stochastic data envelopment analysis method. Section 3 introduces (1) the sources of meteorological data, as well as economic and social data, for the 13 prefecture-level cities in Jiangsu Province, (2) the construction of primary and secondary level indicators affecting PM_2.5_ pollution are provided, (3) three sets of experiments are designed to identify key factors of PM_2.5_ pollution based upon different levels of indicators. Section 4 contains an empirical analysis. Through corresponding analyses and comparisons of the results from the three sets of experiments, the key factors affecting PM_2.5_ pollution are analyzed, and the comprehensive effects of various factors for PM_2.5_ pollution at different risk levels are evaluated. Section 5 is the summary and conclusion.

## 2. Model and Method

Using the chance constrained stochastic DEA method, different experiments were carried out using different levels of indexes. If the result changed substantially after a certain index was deleted, the deleted index had a strong relation with PM_2.5_. Therefore, the following three experiments were designed:

- All 14 secondary indexes were used as input indicators, GDP and PM_2.5_ as output indicators, and chance constrained stochastic DEA was used for the calculation.
- Based on the settings of experiment I, a secondary index was deleted and the remaining 13 input indicators and 2 output indicators were used for the calculation. The purpose of this experiment was to identify which secondary input index has the greatest impact on output indicators.
- As indicators may interact with each other, considering the interrelatedness and mutual influence between indicators, and treating the same type of indexes as one group, we obtained five primary indexes. Based on the settings of experiment I, a primary index was deleted and the remaining four input indicators and two output indicators were used for the calculation. The purpose of this experiment was to identify which primary index has the greatest impact on output indicators.

Data envelopment analysis (DEA) was proposed by the famous operational research scholars, Charnes, Copper and Rhodes, in 1978 [43], and is a non-parametric evaluation method that is relatively efficient when compared to the regression analysis method and least squares method. It is suitable for the evaluation of several decision units with the same type of multiple inputs and outputs. This method does not require determination of the explicit expression of the relationship between input and output variables, which eliminates many subjective factors, and hence, has increased objectivity. Therefore, the DEA method is widely applied in efficiency evaluation.

In this paper, we employ the BCC model: a model for evaluating technical efficiency, named after proposers Banker, Charnes and Cooper in 1984 [44], which is the following optimization problem:

Objective function:

(1) $$\mathrm{Min}\left[\theta -\varepsilon \left(e_{1}{}^{T}S^{-}+e_{2}{}^{T}S^{+}\right)\right]$$

Constraints:

(2) $$\left\{ \begin{array}{l} \sum_{j=1}^{n}X_{j}\lambda_{j}+S^{-}=\theta X_{o}\\ \sum_{j=1}^{n}Y_{j}\lambda_{j}-S^{+}=Y_{o}\\ \sum_{j=1}^{n}\lambda_{j}=1\\ \lambda_{j}\geq 0,j=1,2,\cdots ,n.\\ S^{+}\geq 0\\ S^{-}\geq 0 \end{array}\right.$$

In (2), the input variable of a decision making unit (DMU) in an economic (production) activity is $X=(x_{1},\cdots ,x_{i},\cdots ,x_{m})$, where $x_{i}$ is the *i*-th input;

The output variable is $Y=(y_{1},\cdots ,y_{r},\cdots ,y_{s})$, where $y_{r}$ is the *r*-th output;

$X_{j}$ is the input vector of the j-th DMU, $Y_{j}$ is the output vector of the *j*-th DMU;

$X_{o}$ and $Y_{o}$ are the corresponding indices of the evaluation DMU;

$S^{-}$ is the slack variable of the input variable;

$S^{+}$ is the slack variable of the output variable;

$e_{1}{}^{T}$ is the unit vector in the *m*-th dimension;

$e_{2}{}^{T}$ is the unit vector in the *s*-th dimension;

ε is the Archimedes infinitesimal;

$\lambda_{j}$ is the nonnegative weight of the *j*-th DMU;

Thus, the entire production activity of the DMU can be represented by (X,Y), and the input set of n DMUs can form an n×m matrix, while the output set can form an n×s order output matrix. When the optimum output is 1, i.e., θ=1, the *j*-th DMU is technically effective. When θ<1, the *j*-th DMU is not technically effective.

When dealing with practical problems, input and output variables are often disturbed by random factors such as measurement error and data noise. Because of the random nature of the phenomena and the laws themselves, the input and output variables of DMU may not be accurately determined, and these variables are subject to certain random distribution. There will be great errors when using the traditional DEA model to deal with such problems. In addition, the traditional DEA model has some difficulty in dealing with data with different time scales. Therefore, the chance constrained stochastic DEA method becomes important in practice and can be used to calculate data with different time scales [45].

The chance constrained stochastic DEA model based on the BCC output orientation is the following [46]:

Objective function:

(3) $$\mathrm{Max}\;\theta_{o}$$

Constraints:

(4) $$\left\{ \begin{array}{l} \mathrm{Pr}\left(\sum_{j=1}^{n}\lambda_{j}{\tilde{x}}_{ij}\leq {\tilde{x}}_{io}\right)\geq 1-\alpha ,i=1,2,\ldots ,m\\ \mathrm{Pr}\left(\sum_{j=1}^{n}\lambda_{j}{\tilde{y}}_{rj}\geq \theta_{o}{\tilde{y}}_{ro}\right)\geq 1-\alpha ,r=1,2,\ldots ,s\\ \sum_{j=1}^{n}\lambda_{j}=1\\ \lambda_{j}\geq 0,j=1,2,\ldots ,n \end{array}\right.$$

Pr(.) represents the probability;

α∈[0,1] is the risk level;

$\lambda_{j}$ is the parameter of $DMU_{j}$;

${\tilde{x}}_{ij}$ is the input variables of $DMU_{j}$;

${\tilde{y}}_{rj}$ is the output variable of $DMU_{j}$;

In our study, *n* = 13, representing 13 prefecture-level cities in Jiangsu province. We have m different input variables ${\tilde{x}}_{ij}$, with three different schemes:

(1) m=14, representing the 14 secondary indexes used in Experiment I;
(2) m=13, representing the 13 secondary indexes used in Experiment II;
(3) m=5, representing the 5 primary indexes used in Experiment III.

For output variables ${\tilde{y}}_{rj}$, s=2, representing the 2 output variables.

As Model (2) is an output-oriented model, the reciprocal of the optimal solution of the target value obtained from Model (4) is the stochastic chance constraint efficiency of the evaluated DMU. Model (4) can be rewritten as (without the slack variable):

Objective function:

(5) $$\mathrm{Max}\;\theta_{o}$$

Constraints:

(6) $$\left\{ \begin{array}{l} \sum_{j=1}^{n}\lambda_{j}{\bar{x}}_{ij}-\Phi^{-1}\left(\alpha \right)\sqrt{\sum_{j\neq o}\lambda_{j}^{2}{\left(\sigma_{ij}^{I}\right)}^{2}+{\left(\lambda_{o}-1\right)}^{2}{\left(\sigma_{io}^{I}\right)}^{2}}\leq {\bar{x}}_{io},i=1,2,\ldots ,m\\ \sum_{j=1}^{n}\lambda_{j}{\bar{y}}_{rj}+\Phi^{-1}\left(\alpha \right)\sqrt{\sum_{j\neq o}\lambda_{j}^{2}{\left(\sigma_{rj}^{o}\right)}^{2}+{\left(\lambda_{o}-\theta_{o}\right)}^{2}{\left(\sigma_{ro}^{o}\right)}^{2}}\geq \theta_{o}{\bar{y}}_{ro},r=1,2,\ldots ,s\\ \sum_{j=1}^{n}\lambda_{j}=1\\ \lambda_{j}\geq 0,j=1,2,\ldots ,n \end{array}\right.$$

$\Phi^{-1}\left(\alpha \right)$ is the value of the inverse distribution function of the standard normal distribution function at α;

$\sigma_{i}^{I}\left(\lambda \right)$ is the standard deviation of the linear combination distribution of all i-th input variables;

$\sigma_{ij}^{I}$ and $\sigma_{rj}^{o}$ are the standard deviations of ${\tilde{x}}_{ij}$ and ${\tilde{y}}_{rj}$, respectively;

${\bar{x}}_{ij}$ and ${\bar{y}}_{rj}$ are corresponding mean values of the $DMU_{j}$ input and output.

For the unit to be evaluated DMUo, $\theta_{o}$ in the objective function is the target optimal solution, o∈{1,2,…n}. Let its stochastic efficiency be $\varphi_{o}$, then it holds that φo=1θ.

## 3. Data Sources and Indexes Explanation

In this section, we describe the data collection and data characteristics, as well as indexes, we have constructed.

### 3.1. Data Sources

The meteorological data comes from the China Meteorological Science Data Sharing Service Network (CMSDSSN) ranging from 2013 to 2016 [47]. The CMSDSSN is a meteorological data sharing network system, and a service platform for all domestic and global users.

**Figure 1:**
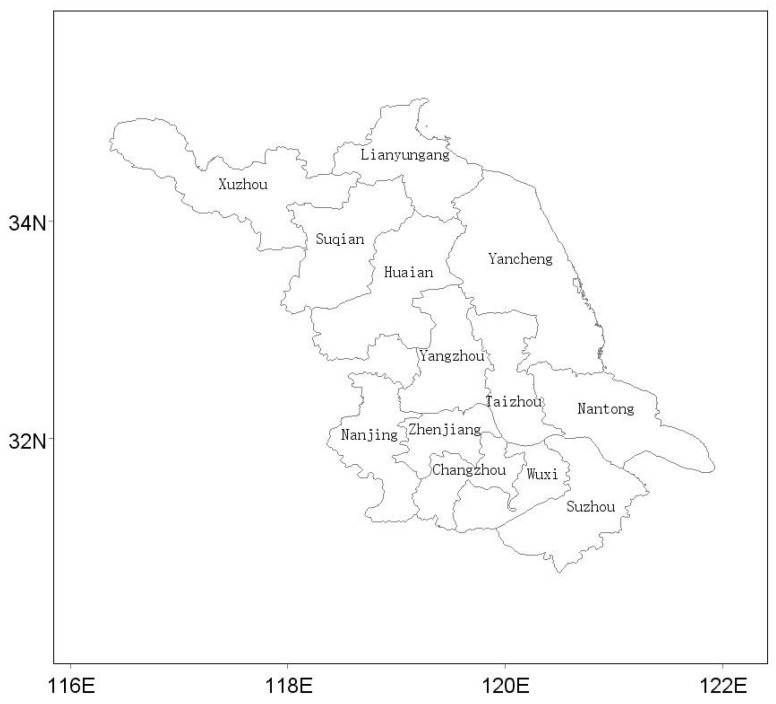
Administrative division map of Jiangsu province.

Social and economic data are derived from the Jiangsu Statistical Yearbook ranging from 2014 to 2017 [48], and statistical yearbook of the 13 prefectural cities (Figure 1) in Jiangsu Province (Xuzhou, Suqian, Lianyungang, Huaian, Yancheng, Yangzhou, Taizhou, Nantong, Zhenjiang, Changzhou, Wuxi, Suzhou, and Nanjing).

Air pollution data comes from the daily monitoring data of atmospheric pollutants from the Environmental Protection Department of Jiangsu ranging from 2013 to 2016. The Jiangsu Environmental Protection Department is responsible for establishing and improving the basic system of environmental protection, monitoring the emission of pollutants.

### 3.2. Indexes Explanation

In this section, five indexes; meteorological conditions, social progress, transportation, energy use and environmental protection, were constructed as primary indexes. Each primary index contained one or more secondary indexes. The setting of the indexes is shown in Table 1. The following are further explanations for the five primary indexes:

Weather conditions refers to different meteorological elements, including six secondary indexes: wind speed, precipitation, temperature, atmospheric pressure, sunshine hours and relative humidity.

Social progress includes the progress and development of material civilization. It is mainly reflected in the progress of material production methods and economic life. It is the achievement of humans in reforming and conquering nature, including improvement of the living standard, improvement of food and clothing, change of life style and many other things. Based on the existing data, three secondary indexes were selected: urbanization rate, population density, and building construction area.

Transportation: Automobile exhaust emissions are one of the major sources of PM_2.5_ and a significant part of air pollutants. While the automobile exhaust monitoring data is not available, two relative indexes from the statistical yearbook were selected: civil car ownership and number of public transportation vehicles in operation.

Energy use: Coal accounts for the majority of all energy use in China, and this particular energy structure makes pollution by coal burning especially serious. It is necessary to study the impact of coal consumption and energy efficiency on air pollution. Two secondary indexes were selected: per 10,000 yuan gross output value of industry energy consumption and total coal consumption.

Environmental protection: The Chinese central government has adopted various policies to reduce PM_2.5_ pollution and damage. Considering the efforts made by people in order to reduce PM_2.5_ pollution, this index was set up. One secondary index was selected: green coverage rate of built-up areas.

Output indexes were divided into two categories: one is the desirable output and the other is the undesirable output. The desirable output is the output that is beneficial to the overall goal and meets expectations, and the undesirable output is a byproduct of the beneficial output.

For economic applications in our setting, larger values mean better economic performance. At the same time, the pollutant discharge should be as low as possible. Therefore, we choose GDP as the desirable output and PM_2.5_ as the undesirable output.

Because the weather data and PM_2.5_ data are collected daily, and the social and economic data from the statistical yearbook is annual data, they need to be pre-processed before the calculation based on the requirements of chance constrained stochastic DEA. For weather data and PM_2.5_ data, the annual mean and variance of data were calculated. For social and economic data, the variance of a sequence of 4-year data was calculated. When the calculation was with the chance constrained stochastic DEA, the variance was the same for 4 years.

## 4. Result Analysis

In this section, we carried out the three designed sets of experiments, analyzed and compared the results from them.

### 4.1. Chance Constrained Stochastic DEA with All 14 Secondary Indexes as Input Indicators Subsection

**Figure 2:**
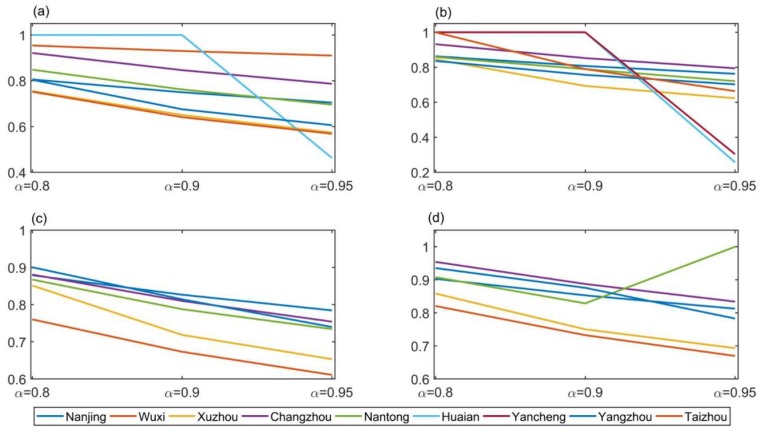
When α is greater than 0.5, the stochastic efficiency of different cities that changed with risk level in (**a**): 2013. (**b**): 2014. (**c**): 2015. (**d**): 2016.

Experiment I was carried out with all 14 secondary indexes as input indicators. In Formula (3), α is the risk level. In this section, we attempt to analyze the difference in the results obtained by selecting different risk levels α, and compare the results obtained in different years with the same risk level α. We took the risk levels α = 0.05, α = 0.1, α = 0.2, α = 0.5, α = 0.8, α = 0.9, and α = 0.95 for the calculation. By using the chance constrained stochastic DEA model, we calculated the target value and stochastic efficiency for the thirteen cities in Jiangsu. The results of all 4 years are shown in Figure 2.

For all 4 years, when the risk level was less than or equal to 0.5, the random efficiency of all cities was 1. When the risk level was greater than 0.5, most cities with changed stochastic efficiency exhibited a decrease in stochastic efficiency with increased risk level.

Table 2 below shows summary results of stochastic efficiency for all 13 cities and the years 2013–2016, with the same risk level in different years. Since the results obtained with different risk levels were highly similar, we used risk level α = 0.95 as an example to illustrate.

From Table 2, we can see that from 2013 to 2016, when the risk level was α = 0.95, the stochastic efficiency of 4 cities including Suzhou, Lianyungang, Zhenjiang and Suqian remained unchanged at 1, the other cities almost all showed increased stochastic efficiency over time, except Suzhou in 2015, Yancheng in 2014, Huaian in 2014 and Taizhou in 2015. The stochastic efficiencies of four cities including Wuxi, Nantong, Huai’an, and Yancheng in 2013 were not 1 and the stochastic efficiencies of 2016 were 1.

### 4.2. Chance Constrained Stochastic DEA with 1 Secondary Index Deleted Each Time

There were 14 input indicators in Experiment I, however, the result of Experiment I cannot distinguish which indicator has the greatest impact on the stochastic efficiency, as seen in Experiment I where all 14 secondary indexes were used. Therefore, experiment II was designed. By deleting 1 secondary index each time, we tried to find which secondary index has the greatest impact on the output indicators.

We took the risk level α = 0.05, α = 0.1, α = 0.2, α = 0.5, α = 0.8, α = 0.9, and α = 0.95 for the calculation. After removing 1 secondary index, the remaining 13 secondary indexes were used as input indicators. Using the chance constrained stochastic DEA model, we calculated the target value and stochastic efficiency of 13 cities in Jiangsu. For α = 0.05, α = 0.1, α = 0.2, α = 0.5 both target value and stochastic efficiency were 1, therefore, these results were omitted. As the results of risk level α = 0.95 was more distinguished than other risk levels (α = 0.8, α = 0.9), we used α = 0.95 as an example to illustrate.

**Figure 3:**
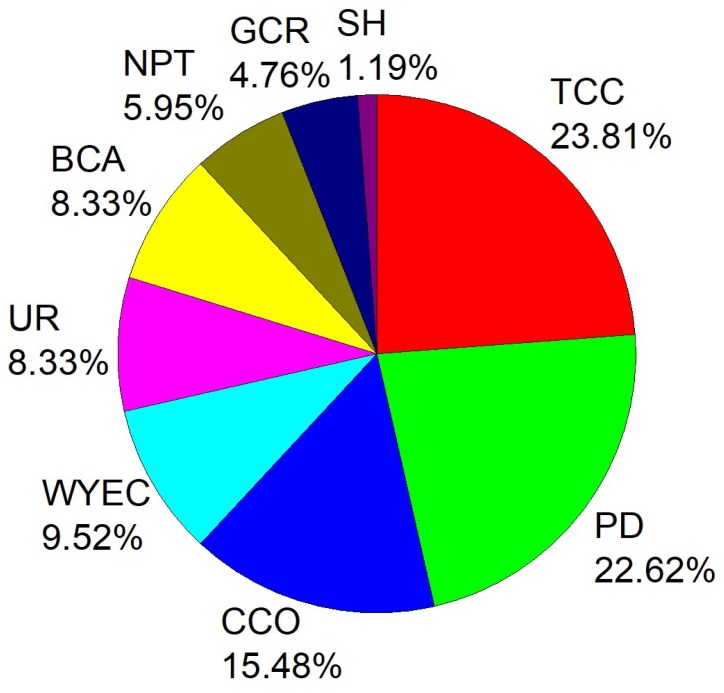
Secondary indexes that changed the stochastic efficiency and their proportion (α = 0.95): total coal consumption (TCC), population density (PD), civil car ownership (CCO), per 10,000 yuan gross output value of industry energy consumption (WYEC), urbanization rate (UR), building construction area (BCA), number of public transportation vehicles under operation (NPT), green coverage rate of built-up area (GCR), sunshine hours (SH).

From Figure 3, it can be seen that for all 4 years, when the risk level was α = 0.95, the change in stochastic efficiency caused by deleting the total coal consumption was the largest, accounting for 23.81% of the changes in stochastic efficiency. The second largest was population density, accounting for 22.62% of the changes in stochastic efficiency. The third largest was civil car ownership, accounting for 15.48% of the changes in stochastic efficiency. The per 10,000 yuan gross output value of industry energy consumption was the fourth, accounting for 9.52% of the changes in stochastic efficiency.

For all 4 years and at a risk level of α = 0.95, when the secondary indexes; wind speed, precipitation, air temperature and atmospheric pressure were deleted, the stochastic efficiency did not change, indicating that these four secondary indexes had no significant effect on the regional GDP and PM_2.5_.

For all secondary indexes that lead to stochastic efficiency changes, removal of human factors accounted for 98.81% of the total changes in stochastic efficiency. Removal of natural causes (sunshine hours) accounted for only 1.19% of the total change. This clearly indicates that human activities play a leading role in PM_2.5_ pollution.

For 2013 alone, secondary indexes that changed the stochastic efficiency were (in order from high to low): total coal consumption, population density, civil car ownership and per 10,000 yuan gross output value of industry energy consumption. This order remained unchanged in the subsequent years 2014–2016.

By comparing the stochastic efficiency between the deleted and non-deleted secondary index for all 14 secondary indexes, and observing whether the stochastic efficiency changed or not, we could tell which secondary index had the greatest impact on the stochastic efficiency. Therefore, when dealing with PM_2.5_ pollution, corresponding policies and measures can be introduced.

### 4.3. Chance Constrained Stochastic DEA with 1 Primary Index Deleted Each Time

As indicators may interact with each other, considering the interrelatedness and mutual influence between indicators, treating the same type of indexes as one group, we get five primary indexes. It is of great significance to examine which primary index has the largest impact on output indicators. Unlike experiment II, experiment III deleted one primary index at a time.

The risk levels α = 0.05, α = 0.1, α = 0.2, α = 0.5, α = 0.8, α = 0.9, and α = 0.95 were used for the calculation. Four primary indexes were used as input indicators after removing the remaining one primary index. The chance constrained stochastic DEA model was used to calculate the target value and stochastic efficiency of 13 cities in Jiangsu. For α = 0.05, α = 0.1, α = 0.2, and α = 0.5, both the target value and stochastic efficiency were 1, therefore, these results were omitted. We used α = 0.95 as an example to illustrate as we did in Figure 3.

**Figure 4:**
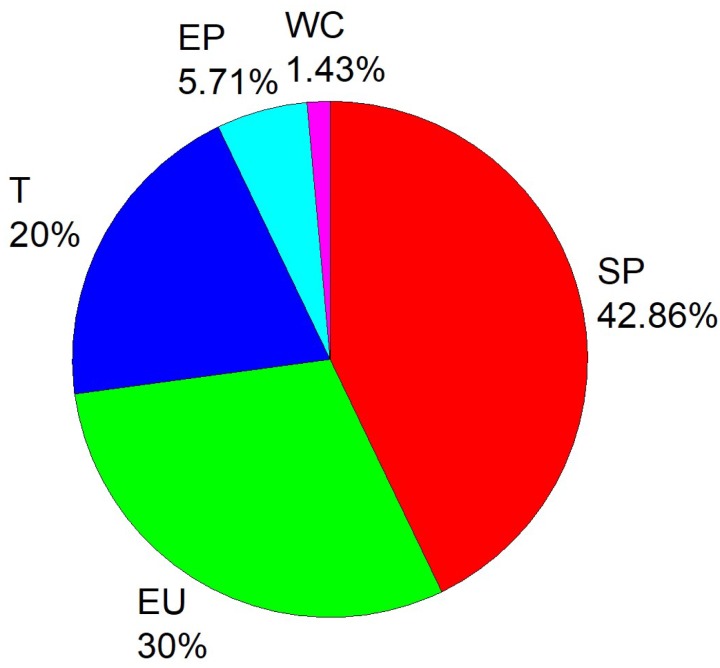
Primary indexes that changed the stochastic efficiency and their proportion (α = 0.95). Social progress (SP), energy use (EU), transportation (T), environmental protection (EP), weather condition (WC).

From Figure 4, it can be seen that for all 4 years, when the risk level was α = 0.95, the change in stochastic efficiency caused by deleting the social progress index was the largest, accounting for 42.86% of the changes in stochastic efficiency. The second largest was the energy use index, accounting for 30.00% of the changes in stochastic efficiency. The third largest was the transportation index, accounting for 20.00% of the changes in stochastic efficiency.

For all primary indexes that changed stochastic efficiency, the removal of the weather condition index accounted for only 1.43% of the total change in stochastic efficiency. Removal of the human factor index accounted for 98.57% of the total change in stochastic efficiency, meaning that human activities are more associated with PM_2.5_ pollution. Hence, human activities are the main reason for PM_2.5_ pollution.

For 2013 alone, primary indexes that changed the stochastic efficiency were (in order from high to low): social progress, energy use and transportation, which remained unchanged in the subsequent years from 2014 to 2016.

Now we summarize results from all three experiments:

(1) For the same year, the stochastic efficiency decreased with an increased risk level. For the same risk level, the stochastic efficiency increased with later years.
(2) By deleting one secondary index at a time, we determined the secondary index (total coal consumption) that had the greatest impact on output indicators. The second largest was population density, the third largest was civil car ownership, and per 10,000 yuan gross output value of industry energy consumption was the fourth. Removal of human factors accounted for 97.94% of the total changes in stochastic efficiency. Removal of natural causes (wind speed and sunshine hours) accounted for only 2.06% of the total change. While some meteorological elements such as wind speed and temperature do have a large impact on PM_2.5_, we used annual data in this study and did not show this. Daily or hourly data will be more likely to show the importance of meteorological elements.
(3) By deleting one primary index at a time, we found the primary index (social progress) that had the greatest impact on output indicators. The second largest was energy use, and the third largest was transportation. Removal of the human factor index accounted for 97.51% of the total changes in stochastic efficiency while removal of the weather condition index accounted for only 2.49% of the total change.
(4) Human activities are deeply associated with PM_2.5_ pollution and the impact of meteorological factors is far less than that caused by human activities.

## 5. Discussion

One of the main drawbacks of the traditional DEA method is that it cannot handle measurement errors. Traditional DEA is very sensitive to data errors because efficient units determine the efficiency [49]. Therefore, it is very important to incorporate random variations into DEA analysis. Compared with traditional DEA, chance constrained stochastic DEA has the following two advantages: it admits random input and output, and allows the evaluation unit to exceed the front edge under the given probability constraint.

In developed countries, people have experienced severe haze pollution during the industrialization era, e.g., the Los Angeles photochemical smog incident [50], the Donora smoke event [51], the great smog of London [52] and the Meuse valley fog [53]. These pollution incidents caused huge damage to people’s health and personal property. These pollution incidents were controlled through targeted measures and persistent governance [54]. In China, the Central Government took various measures to lower the level of haze pollution and reduce the damage caused by haze. On 29 February 2012, the government issued a newly revised “ambient air quality standard” that established a standard to dynamically monitor the haze pollution and officially release timely real time air quality indexes. For the first time, PM_2.5_ was added to the national environmental quality standard. Since then, China’s PM_2.5_ pollution has been significantly reduced. The annual average concentration of PM_2.5_ decreased from 72 μg/m^3^ in 2013 to 47 μg/m^3^ in 2016.

## 6. Conclusions

In this paper, we applied the chance constrained stochastic DEA to explore the relationship between PM_2.5_ pollution and meteorological elements, along with human activities, by analyzing the meteorological data, daily air pollutants monitoring data, and social and economic data derived from the Jiangsu Statistical Yearbook and the Statistical Yearbook of 13 prefectural cities in Jiangsu Province. The results showed that chance constrained stochastic DEA can clearly identify that human activities are the main cause of PM_2.5_ pollution in Jiangsu Province. Chance constrained stochastic DEA is rarely used to assess PM_2.5_ pollution. Our research results show that this method is very suitable for evaluating the PM_2.5_ pollution problem.

## Figures and Tables

**Table 1:** Description of indexes.

Variable	Primary Index	Secondary Index	Definition
Input	Weather Condition	Wind Speed	The velocity of air relative to a fixed place on the earth.
		Precipitation	The amount of precipitation in a region.
		Temperature	The degree of air cooling and heating.
		Atmospheric Pressure	Force per unit area exerted by an atmospheric column.
		Sunshine Hours	Duration of sunshine in a day.
		Relative Humidity	The percentage of water vapor pressure to the saturated vapor pressure in the air.
	Social Progress	Urbanization Rate	The percentage of the total population living in urban areas.
		Population Density	Number of people per square kilometer.
		Building Construction Area	Total construction area of the buildings constructed during the reporting period.
	Transportation	Civil Car Ownership	Vehicles registered under civil vehicle licenses.
		Number of Public Transportation Vehicles under Operation	Public transportation vehicles that serve residents, different vehicles are converted to the same standard.
	Energy Use	Per 10,000 Yuan Gross Output Value of Industry Energy Consumption	The percentage of energy consumption by enterprises to the total industrial output value.
		Total Coal Consumption	The amount of coal consumed converted into the amount of standard coal.
	Environmental Protection	Green Coverage Rate of Built-up Area	The percentage of green coverage area to built-up area.
Output	Expected Output	Gross Regional Product	The market value of all final goods and services produced in a region.
	Undesirable Output	PM_2.5_	Concentration of particles in air with diameters less than or equal to 2.5 microns.

**Table 2:** Stochastic efficiency of 13 cities in Jiangsu Province from 2013 to 2016 when risk level α = 0.95.

City	2013	2014	2015	2016
Nanjing	0.7048	0.763	0.7845	0.8127
Wuxi	0.9102	1	1	1
Xuzhou	0.574	0.6234	0.6533	0.6933
Changzhou	0.787	0.7945	0.7542	0.8341
Suzhou	1	1	1	1
Nantong	0.6958	0.7211	0.7342	1
Lianyungang	1	1	1	1
Huaian	0.4629	0.2572	1	1
Yancheng	1	0.3037	1	1
Yangzhou	0.6061	0.7027	0.7398	0.7829
Zhenjiang	1	1	1	1
Taizhou	0.5685	0.664	0.6111	0.6697
Suqian	1	1	1	1
